# King Edward VII.'s Hospital, Cardiff

**Published:** 1911-09-23

**Authors:** 


### "KING EDWARD VII.'S HOSPITAL, CARDIFF."

The authorities of the Cardiff Infirmary, as we note in "Week's Work," have received his Majesty King George's gracious consent that the institution may in future be called "King Edward VII.'s Hospital, Cardiff," which was conveyed to the Board of Management at their meeting this month in the following communication:

Home Office, Whitehall,
September 8, 1911.

Sir,—I am directed by the Secretary of State to acquaint you, for the information of the Board of Management of the Cardiff Infirmary, that the King has been graciously pleased to approve of the Institution being styled "King Edward VII.'s Hospital, Cardiff."

With regard to the Board's desire that the King should open the new wing of the Infirmary, I am to say that no decision can be given until next year. The Board may then perhaps submit the proposal again to his Majesty's Private Secretary.—I am, Sir, your obedient servant,

(Signed) W. P. Byrne.

Leonard D. Rea, Esq.
The Infirmary, Cardiff.

We are asked to make clear to the public generally, though it will not be necessary to remind those who are intimate with the good work of the Cardiff Infirmary, that the institution dates back to 1837, and the new name must not be permitted to suggest that the hospital has been instituted during the reign of his late Majesty. With this reservation, no title could be more appropriate, for it is within his late Majesty's reign that the Cardiff Infirmary has proved itself capable of developing into a first-class modern hospital, and the efficiency of the institution and its phenomenal extensions of the past decade may be traceable to the inspiration of King Edward's reasoned sympathy with all that is best in modern hospital development.

Moreover, the working people of Cardiff and South Wales—of whom the warm-hearted colliers are probably the most numerous class—have it within their power, in co-operation with the wealthy people of the district, to give a substantial significance to the new title, by assuring that the hospital, now identified with the beloved name of his late Majesty, is assured against the uncertainties of spasmodic support. This will be achieved by the accomplishment of the second object of the Cardiff City Memorial to King Edward VII. —namely, the addition of £100,000 to the invested capital of the institution. It may be confidently hoped that his Majesty King George's gracious message will strengthen the hands of Colonel Bruce Vaughan in the vigorous appeals which he addresses, from time to time, to the South Wales community, and that if the King should open the new wing of the infirmary next year—as may be reasonably anticipated from the foregoing letter—his Majesty will find that the first hospital of the Principality, and one which now bears the illustrious name of his Royal father, has been assured a maintenance in harmony with the trust implied by his Majesty's recognition and with the urgent requirements of the wide industrial area, at present—entirely owing to financial considerations—but inadequately served by the Cardiff Infirmary.

